# Inhibitory effects of H-Ras/Raf-1-binding affibody molecules on synovial cell function

**DOI:** 10.1186/s13568-014-0082-3

**Published:** 2014-11-11

**Authors:** Seiji Shibasaki, Miki Karasaki, Torbjörn Gräslund, Per-Åke Nygren, Hajime Sano, Tsuyoshi Iwasaki

**Affiliations:** General Education Center, Hyogo University of Health Sciences, 1-3-6 Minatojima, Chuo-ku, Kobe, 650-8530 Japan; Graduate School of Pharmacy, Hyogo University of Health Sciences, 1-3-6 Minatojima, Chuo-ku, Kobe, 650-8530 Japan; Division of Rheumatology, Department of Internal Medicine, Hyogo College of Medicine, 1-1Mukogawa-cho, Nishinomiya, 663-8501 Japan; Department of Molecular Biotechnology, School of Biotechnology, Royal Institute of Technology (KTH), AlbaNova University Center, Stockholm, SE-106 91 Sweden; Division of Pharmacotherapy, Department of Pharmacy, School of Pharmacy, Hyogo University of Health Sciences, 1-3-6 Minatojima, Chuo-ku, Kobe, 650-8530 Japan

**Keywords:** Affibody molecules, Inflammation, Interleukin-6, Intracellular signal transduction, Prostaglandin E2, Rheumatoid arthritis

## Abstract

Affibody molecules specific for H-Ras and Raf-1 were evaluated for their ability to inhibit synovial cell function. Affibody molecules targeting H-Ras (Z_ras122_, Z_ras220_, and Z_ras521_) or Raf-1 (Z_raf322_) were introduced into the MH7A synovial cell line using two delivery methods: transfection with plasmids encoding the affibody molecules or direct introduction of affibody protein using a cell-penetrating peptide reagent. Interleukin-6 (IL-6) and prostaglandin E2 (PGE2) production by MH7A cells were analyzed by enzyme-linked immunosorbent assay after stimulation with tumor necrosis factor-alpha (TNF-α). Cell proliferation was also analyzed. Phosphorylation of extracellular signal-regulated kinase (ERK) was analyzed by western blot. All affibody molecules could inhibit IL-6 and PGE2 production in TNF-α-stimulated MH7A cells. The inhibitory effect was stronger when affibody molecules were delivered as proteins via a cell-penetrating peptide reagent than when plasmid-DNA encoding the affibody moelcules was transfected into the cells. Plasmid-expressed Z_ras220_ inhibited phosphorylation of ERK in TNF-α-stimulated MH7A cells. Protein-introduced Z_raf322_ inhibited the production of IL-6 and PGE2 and inhibited cell proliferation in MH7A cells. These findings suggest that affibody molecules specific for H-Ras and Raf-1 can affect intracellular signal transduction through the MAP kinase pathway to inhibit cell proliferation and production of inflammatory mediators by synovial cells.

**Electronic supplementary material:**

The online version of this article (doi:10.1186/s13568-014-0082-3) contains supplementary material, which is available to authorized users.

## Introduction

Rheumatoid arthritis (RA) is an inflammatory disease characterized by the progressive destruction of joints. In RA, hyperplasia of synovial lining cells is observed along with angiogenesis and inflammatory mononuclear cell infiltration (Kitano et al. [2006]). A wide variety of molecules produced by both synovial cells and infiltrating inflammatory mononuclear cells is thought to be involved in RA pathology. Specifically, proinflammatory cytokines such as interleukin-6 (IL-6), IL-17 (Schlegel et al. [2013]) and tumor necrosis factor alpha (TNF-α) play a key role in RA pathogenesis, including the activation of synovial fibroblasts, osteoclasts, and inflammatory mononuclear cells, which results in irreversible damage to the soft tissues and bones (Olsen and Stein [2004]).

One of the conventional disease-modifying antirheumatic drugs (DMARDs), methotrexate, is the standard for RA pharmacotherapy (Upchurch and Kay [2012]). However, its treatment outcomes are not satisfactory and often cause high levels of toxicity and adverse effects for patients (Smolen and Steiner [2003]). In recent years, various biological agents have been developed and used clinically as important options for RA treatment (Saag et al. [2008]). These biological agents include an anti-CD20 antibody (rituximab) (Mélet et al. [2013]), TNF antagonists (e.g., adalimumab, etanercept, golimumab, and infliximab), an IL-6 receptor antagonist (tocilizumab) (Smolen and Aletaha [2011]), and a recombinant fusion protein consisting of the extracellular domain of human CTLA-4 (abatacept) (Gómez-Reino [2012]). Although these biological agents provide favorable treatment outcomes for RA patients, in view of medical economics, biological therapies are more costly than traditional DMARDs. Thus, a more cost-effective treatment option is desirable because healthcare payers and providers are conscious of cost in light of the current period of budget constraints (Furneri et al. [2012]).

Several biologically active, non-immunoglobulin-based proteins have been created as candidates of novel biological agents. For instance, a monobody is a target-binding protein that is generated from the fibronectin type III domain (Koide et al. [2012]). Synthetic ankyrin repeat proteins have a binding scaffold and are potential diagnostic and therapeutic tools (Baumann et al. [2007]). In this study, we investigated another class of non-immunoglobulin ligands, referred to as affibody molecules. They are engineered binding proteins based on a scaffold of the three-α-helix bundle Z-domain (Friedman and Ståhl [2009]; Nilsson and Tolmachev [2007]; Nygren [2008]). The 6-kDa Z-domain is derived from the B-domain, one of the five domains in *Staphylococcus aureus* protein A (Gunneriusson et al. [1996]).

Affibody molecules associated with signal transduction or transcription in tumor development or immune responses have been screened and applied. For example, affibody molecules against c-Jun, an oncogenic transcription factor, have been selected and are able to detect c-Jun in a melanoma cell line (Lundberg et al. [2009]). Furthermore, other affibody molecules against human epidermal growth factor receptor (HER), specifically HER2 (Lindberg et al. [2012]) and HER3 (Malm et al. [2013]), have been investigated.

We have previously reported the selection of H-Ras-binding or Raf-1-binding affibody molecules (Grimm et al. [2010]). Affibody clones Z_ras122_, Z_ras220_, and Z_raf322_ were isolated by phage display selection from a library, and the specificity and affinity for H-Ras or Raf-1 proteins were determined in that study. Each molecule was demonstrated to selectively bind Ras or Raf-1 proteins, but not other human control proteins. Z_ras122_, Z_ras220_, and Z_raf322_ had binding affinities in the high nanomolar to low micromolar range, as demonstrated by a real-time biospecific interaction analysis. As an alternative or complement to current RA treatment regimens, these affibody molecules might have the potential to inhibit signal transduction mediated by Ras and Raf, since this signaling is thought to be important for efficient synthesis of several inflammatory mediators (Yamamoto et al. [2003]).

In this study, we investigated the effect of introducing H-Ras-binding and Raf-1-binding affibody molecules in the MH7A synovial cell line (Tsunemi et al. [2010]). First, we subcloned DNA encoding affibody molecules against H-Ras or Raf-1 into a plasmid DNA vector for mammalian expression, and the vectors were introduced into MH7A cells. Then, affibody proteins were produced using an *Escherichia coli* expression system and were introduced into MH7A cells. We investigated inhibition caused by H-Ras- or Raf-1-targeting affibody molecules on the production of the inflammatory mediators IL-6 and prostaglandin E2 (PGE2). Furthermore, we examined proliferation of MH7A cells.

## Materials and methods

### Cell line

The human MH7A synovial cell line (Riken, Saitama, Japan), which originated from intra-articular soft tissue of the knee joints of an RA patient, was established by transfection with the SV40 T antigen (Miyazawa et al. [1998]). MH7A cells were cultured in RPMI 1640 (Sigma, St. Louis, MO, USA) containing 10% heat-inactivated fetal bovine serum (Whittaker, Walkersville, MD, USA), 100 units/mL of penicillin, and 100 μg/mL of streptomycin (Invitrogen, CA, USA) at 37°C in an atmosphere of 5% CO_2_ in air.

### Plasmid construction for affibodies and introduction into MH7A cells

The plasmids that were used to produce affibodies in mammalian cells were constructed by first amplifying DNA encoding anti-H-Ras and anti-Raf-1 affibodies (Z_ras122_, Z_ras220_, Z_ras521_, and Z_raf322_) by PCR using primers 5'-AAGGGGATCCACCATGGGCAGCAGCCATCATCATCA-3' and 5'-AGGGGTTATGCTAGTTATTGCTCAGCGCGGAATTCTTA-3' (Grimm et al. [2010]). DNA sequences of affibody molecules are listed the additional file (Additional file 1). Each fragment was inserted into the multiple cloning site of pcDNA3.1(+) (Invitrogen). The nucleotide sequences of these constructs were confirmed by sequencing with an ABI PRISM 3100 Genetic Analyzer (Applied Biosystems, Foster City, CA, USA). Constructs were introduced into MH7A cells for transient expression using Lipofectamine 2000 (Invitrogen) following the manufacturer's instructions. Cells used for transfection were seeded at 1.7 × 10^5^ cells/well in a 24-well plate (BD, Franklin Lakes, NJ, USA) and cultured for 1 d before transfection.

### Western blot analysis of affibody expression

MH7A cells transfected with affibody-encoding plasmids (1 × 10^7^) were plated in a 24-well plate. After 48 or 72 h of incubation, cells were lysed in Mammalian Lysis Buffer from the Qproteome Mammalian Protein Prep Kit (Qiagen, Hilden, Germany), and protein content was determined using the Bio-Rad Protein Assay Reagent (Bio-Rad, Hercules, CA, USA) with bovine serum albumin as the standard. Each sample was resolved by SDS-PAGE on 20% polyacrylamide gels and then transferred to 0.45-μm nitrocellulose membranes. After blocking overnight at 4°C with Blocking One Solution (Nacalai Tesque, Kyoto, Japan), membranes were incubated with goat polyclonal antibody specific for the affibody (Abcam, Cambridge, UK) as the primary antibody (1:400 dilution in phosphate-buffered saline; PBS) overnight at 4°C. After washing the membranes with Tris-buffered saline-0.05% Tween 20 (washing buffer), target proteins were detected with alkaline phosphatase (AP)-labeled ReserveAP Rabbit anti-goat immunoglobulin G (IgG) (KPL, Gaithersburg, MD, USA; 1:1,000 dilution in 1% PBS containing 0.05% Tween 20). NBT/BCIP (Roche, Mannheim, Germany) was used as the AP colorimetric substrate for visualization.

### Measurement of IL-6 or PGE2 levels

MH7A cells (2 × 10^4^) that were transfected with an affibody-encoding plasmid were plated in a 24-well plate and cultured in the presence or absence of 100 ng/mL TNF-α. After 24 or 48 h of incubation, IL-6 and PGE2 levels were determined by assaying supernatants with an Enzyme-Linked Immunosorbent Assay (ELISA) Kit for IL-6 (R&D Systems, Minneapolis, MN, USA) or PGE2 (ENZO Life Sciences, Farmingdale, NY, USA), respectively, according to the instructions.

### Cell proliferation studies

Cell viability was determined using a Cell Counting Kit (CCK)-8 (Dojindo, Kumamoto, Japan), in which 2-(2-methoxy-4-nitrophenyl)-3-(4-nitrophenyl)-5-(2,4-disulfophenyl)-2H-tetrazolium monosodium salt was used as a substrate (Ohuchida et al. [2004]). Absorbance was measured using a SpectraMax M2 Microplate Reader (Molecular Devices, Sunnyvale, CA, USA) at 450 nm (Shibasaki et al. [2012]).

### Western blot analysis for phosphorylated extracellular signal-regulated kinase (p-ERK)

Western blot analysis was performed as described above. Briefly, MH7A cells transfected with affibody-encoding plasmids (1 × 10^7^) were plated in a 24-well plate, incubated for 24 h, and then stimulated with 100 ng/mL TNF-α (Sigma). After an additional incubation of 16 or 24 h, the soluble fraction of the cells was analyzed by western blot analysis using IgG against p-ERK (Santa Cruz Biotechnology, Dallas, TX, USA) as a primary antibody. Goat polyclonal IgG against actin (Santa Cruz) was used as a control.

### Affibody protein production in *E. coli*

Expression of the pAffi-Z_ras122_, -Z_ras220_, -Z_ras521_, and -Z_raf322_ plasmids in *E. coli* BL21(DE3) produced H-Ras and Raf-1 affibody molecules with an N-terminal 6 × His tag. The *E. coli* cells were inoculated in 2 mL of Luria-Bertani (LB) broth containing 100 μg/L ampicillin and were incubated in a shaking incubator overnight at 37°C. The 2-mL overnight cultures were used to inoculate fresh LB medium containing 100 μg/L ampicillin (125 mL), and cultures were grown at 37°C to an OD_600_ of 0.6-0.7. Gene expression was then induced by the addition of isopropyl β-D-thiogalactoside (Wako Pure Chemical, Osaka, Japan) to a final concentration of 1 mM. After 3-4 h of incubation at 37°C, the cells were harvested by centrifugation (4,000× *g*, 15 min, 4°C). The cell pellets were subsequently resuspended in 10 mL of B-PER Bacterial Protein Extraction Reagent (Thermo Fisher Scientific, Waltham, MA, USA) and shaken gently for 10 min at room temperature (25°C). Cell debris was pelleted by centrifugation at 27,000 × *g* for 20 min. Supernatants containing soluble proteins were purified by recovering the 6 × His-Z_ras122_, -Z_ras220_, -Z_ras521_, and -Z_raf322_ fusion proteins after passage through nickel-chelated agarose columns (Thermo Fisher Scientific). The columns were equilibrated with 10 mL of B-PER Bacterial Protein Extraction Reagent before application of the supernatant. After washing the column with B-PER wash buffer, the bound proteins were released with elution buffer (50 mM Tris, 300 mM NaCl, 200 mM imidazole, 10% [v/v] glycerol), according to a previous protocol (Shibasaki et al. [2013]). The concentration of each protein was determined by the BCA assay (REF), and the molecular size was confirmed by SDS-PAGE.

### Affibody protein introduction into MH7A cells

MH7A cells were seeded at 1.7 × 10^5^ cells/well in a 24-well plate and cultured for 1 d before the introduction of the purified proteins. Each affibody protein (5 μg) was mixed with Xfect Protein Buffer (Takara Bio, Shiga, Japan) to a total volume to 20 μL and then mixed with Xfect Protein Transfection Reagent. As a negative control, PBS was used in the place of the affibody molecule. After substitution of culture medium with serum-free medium for the cells, a diluted protein mixture was added to cultured MH7A cells and incubated at 37°C, according to a previous protocol (Kato et al. [2013]).

### Statistical analysis

Results are expressed as the mean ± SD. The significance of the difference between the experimental results and control values was determined by Student's *t*-test. *P* values less than 0.05 were considered significant.

## Results

### Expression of affibody molecules in MH7A cells

**Figure 1:**
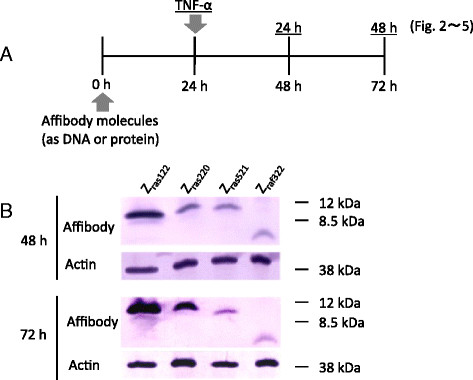
**Experimental protocol (A) and western blot analysis for confirmation of cytosolic synthesis of affibody molecules (B). (A)** Affibody molecules were introduced into MH7A cells encoded by DNA or as proteins. After 24-h culture, the cells were stimulated with TNF-α (100 ng/mL) and cultured for an additional 24-48 h. **(B)** Western blot analysis of each soluble fraction from MH7A cells harboring plasmid-encoded affibody protein molecules was analyzed at 48 and 72 h after the introduction of the plasmid into MH7A cells. Actin was analyzed as a control.

Each pcDNA3.1-based plasmid encoding Z_ras122_, Z_ras220_, or Z_ras521_ was designed to yield head-to-tail homodimers of the molecules. The plasmid encoding Z_raf322_ was designed to yield a monomeric molecule. Each plasmid was introduced into MH7A cells, and protein expression was confirmed by western blot analysis. The dimeric affibody molecules, Z_ras122_, Z_ras220_, and Z_ras521_, appeared to be approximately 12 kDa, and the monomeric affibody molecule, Z_raf322_, appeared to be approximately 6 kDa. The Z_ras122_ affibody protein was expressed in a greater quantity than the other affibody proteins at 48 and 72 h after transfection (Figure 1).

### Inhibition of IL-6 and PGE2 production by plasmid-expressed affibody proteins

**Figure 2:**
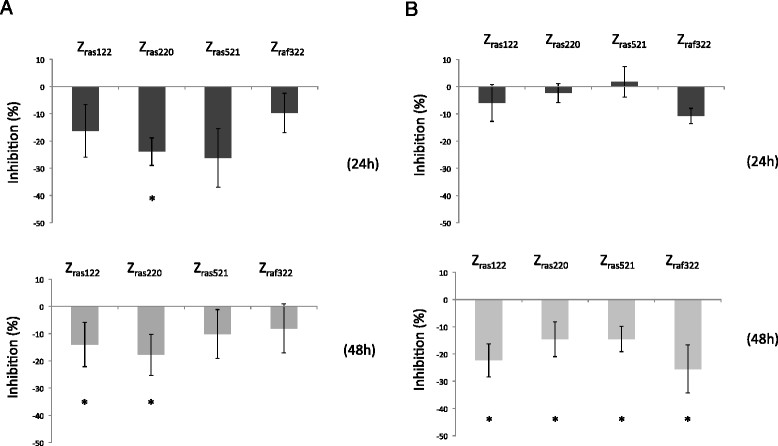
**IL-6 (A) and PGE2 (B) production by MH7A cells with plasmid-expressed affibodies.** MH7A cells were transfected with plasmids encoding affibodies. After 24 h of culture, the cells were stimulated with TNF-α (100 ng/mL) and cultured for an additional 24-48 h. Data represent the percent inhibition of IL-6 or PGE2 production by MH7A cells. Percent inhibition was calculated as follows: ([(IL-6 or PGE2 levels in MH7A cells transfected with affibody DNA) - (IL-6 or PGE2 levels in MH7A cells transfected with pcDNA3.1)]/[IL-6 or PGE2 levels in MH7A cells transfected with pcDNA3.1]) × 100. Data represent the means × SD of three independent experiments. *p <0.05.

Each plasmid expressing the affibody molecules was introduced into MH7A cells to observe any inhibition of IL-6 production. At 24 h after transfection, TNF-α was added to induce synthesis of IL-6 and PGE2 (Figure 1A). After an additional 24-48 h of culture, the supernatant was collected, and IL-6 production was evaluated by ELISA. Z_ras220_ reduced IL-6 production by approximately 20% relative to the empty plasmid (pcDNA3.1) at 24 and 48 h after TNF-α stimulation; in contrast, expression of Z_raf322_ reduced IL-6 production by approximately 10% (Figure 2A). The inhibition of IL-6 production by each plasmid-expressed affibody was greater at 24 h than at 48 h after TNF-α stimulation (Figure 2A). Possible inhibition of PGE2 production in MH7A cells was also analyzed upon expression of the affibody molecules. Expression of Z_raf322_ reduced PGE2 production by approximately 10% relative to the empty plasmid at 24 h after TNF-α stimulation, while expression of Z_raf122_. Z_ras220_, and Z_ras521_ did not significantly affect PGE2 expression at 24 h (Figure 2B). Z_ras122_ and Z_raf322_ reduced PGE2 production by approximately 20% relative to the control plasmid at 48 h after TNF-α stimulation; in contrast to IL-6 production, the inhibition of PGE2 production by each plasmid-expressed affibody was greater at 48 h than at 24 h after TNF-α stimulation (Figure 2B).

### Phosphorylation of ERK

**Figure 3:**
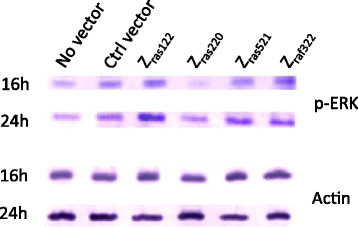
**Western blot analysis for the confirmation of ERK1/2 phosphorylation.** MH7A cells were transfected with plasmids encoding affibodies. After 24 h of culture, the cells were stimulated with TNF-α (100 ng/mL) and cultured for an additional 24-48 h. Each soluble fraction of MH7A cells harboring plasmid-encoded affibody molecules was analyzed via western blot for the expression of p-ERK and actin at 16 and 24 h after TNF-α stimulation. Representative data are shown for p-ERK and actin expression by MH7A cells.

ERK1/2 is situated downstream of Ras/Raf in the MAP kinase signal transduction cascade. To determine if affibody molecules affected signaling through this pathway, phosphorylation of ERK1/2 was evaluated in MH7A cells at 16 and 24 h after TNF-α stimulation. Western blot analysis of each cell lysate was performed, using an anti-p-ERK antibody with an anti-actin antibody serving as a control. The expression of Z_ras220_ inhibited phosphorylation of ERK1/2 at 16 h after TNF-α stimulation (Figure 3). This result indicated that Z_ras220_, but not the other affibody proteins, was able to block ERK1/2 phosphorylation through binding to Ras.

### Inhibition of IL-6 and PGE2 production by affibody molecules as proteins in MH7A cells

**Figure 4:**
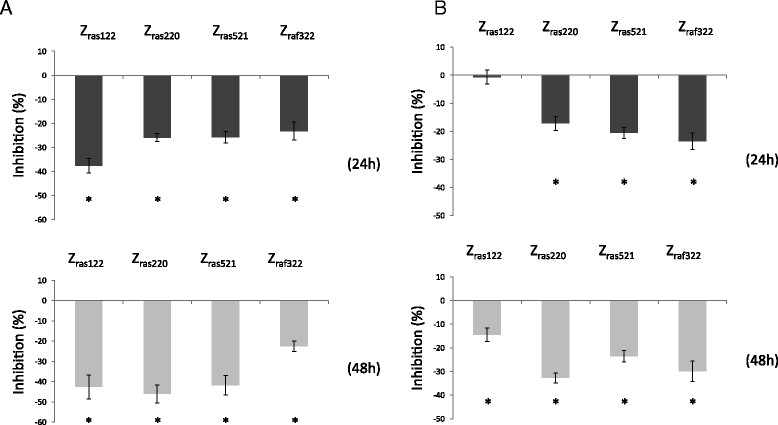
**IL-6 (A) and PGE2 (B) production by affibody protein-introduced MH7A cells.** MH7A cells were transfected with affibody proteins. After 24 h of culture, the cells were stimulated with TNF-α (100 ng/mL) and cultured for an additional 24-48 h. IL-6 and PGE2 levels were analyzed, and percent inhibition of IL-6 or PGE2 production was determined. Percent inhibition was calculated as follows: ([(IL-6 or PGE2 levels in affibody protein-introduced MH7A cells) - (IL-6 or PGE2 levels in MH7A cells introduced with standard peptides)]/([IL-6 or PGE2 levels in MH7A cells introduced with standard peptides]) × 100. Data represent the means × SD of three independent experiments. *p <0.05.

The affibody molecules were expressed in *Escherichia coli* and purified to >95% purity. SDS-PAGE analysis of the fractions from the last chromatographic step revealed essentially pure proteins of the expected molecular weight (data not shown). Individually, Z_ras122_, Z_ras220_, Z_ras521_, and Z_raf322_ proteins were introduced into MH7A cells by incubation with the cell-penetrating peptide reagent Xfect. Although the inhibition of IL-6 production by Z_ras122_ was stronger than the inhibition by the other proteins at 24 h, Z_ras220_ protein strongly inhibited IL-6 production by approximately 46% relative to the control penetrating peptide alone at 48 h after TNF-α stimulation (Figure 4A). The Z_ras220_ protein also strongly inhibited PGE2 production by 32% relative to the control penetrating peptide alone at 48 h after TNF-α stimulation (Figure 4B).

### Effect of affibody molecules on cell proliferation

**Figure 5:**
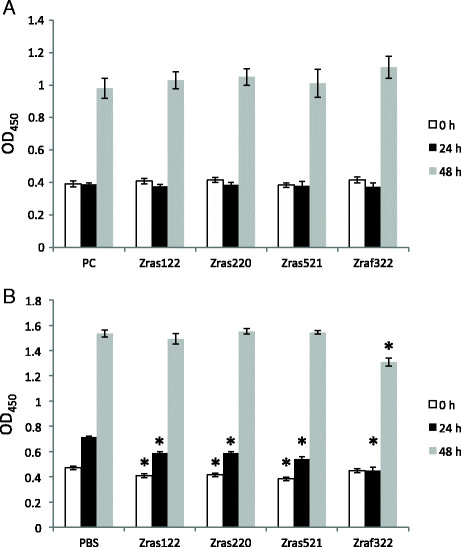
**Cell proliferation assay for MH7A cells transfected with plasmid-expressed affibody molecules (A) and MH7A cells introduced affibody molecules as protein (B).** After 24 h of culture, the cells were stimulated with TNF-α (100 ng/mL) and cultured for an additional 24-48 h. Cell proliferation was analyzed with a CCK assay. Data represent the means × SD of three independent experiments. *p <0.05.

The proliferation of MH7A cells was also investigated upon expression of the affibody molecules by plasmid transfection. No change in proliferation could be observed at 24 or 48 h after TNF-α stimulation for any of the constructs, compared to the cells harboring the empty plasmid (Figure 5A). We next investigated a possible effect on MH7A cell proliferation after introduction of affibody proteins. MH7A cells transfected with a control peptide proliferated 151% from 0-24 h, while those transfected with Z_raf322_ proteins proliferated almost 0% from 0-24 h (Figure 5B). Furthermore, during 0-48 h, control cells and Z_raf322_ proteins-transfected cells proliferated 327% and 291%, respectively. These results indicate an inhibitory effect of Z_raf322_ protein introduction on proliferation.

## Discussion

In this study, we investigated whether affibody molecules targeting H-Ras or Raf-1 can inhibit cell proliferation and the production of inflammatory mediators *in vitro*. To date, it has been demonstrated that intracellularly expressed affibody molecules can bind to target molecules and inhibit their functions. For example, an affibody molecule that has an affinity for epidermal growth factor receptor (EGFR) retains EGFR in the secretory compartment. This constraint led to decreased phosphorylation of EGFR and a reduced proliferation rate (Vernet et al. [2009]). An affibody molecule targeting HER-3 inhibits proliferation of a cancer cell line (Göstring et al. [2012]). However, we demonstrated for the first time that an affibody targeting intracellular signal transduction molecules could inhibit cell proliferation and the production of inflammatory mediators. We demonstrated that H-Ras- (Z_ras122_, Z_ras220_, Z_ras521_) or Raf-1- (Z_raf322_) targeting affibody molecules inhibited the production of IL-6 and PGE2 in a synovial cell line. These affibody molecules were introduced into the cells via DNA-encoding plasmids or as purified proteins. In both cases, inhibition of IL-6 and PGE2 production was observed for almost all affibody molecules.

Several previous reports indicated suppression of synovial cell function by Ras inhibition. For instance, gene transfer of a dominant negative mutant of the *Ras* gene (Yamamoto et al. [2003]) or gene silencing using a locked nucleic acid against Ras (de Launay et al. [2010]) in synovial cells inhibited the production of inflammatory mediators, such as IL-6. Consistent with these reports, we observed that affibody molecules binding to H-Ras or Raf-1 could inhibit the production of IL-6. PGE2 production by RA synovial cells is regulated by Ras (Na et al. [2004]). Therefore, we analyzed PGE2 levels and demonstrated that these affibody molecules also inhibited PGE2 production. Matrix metalloproteinase (MMP)-3 is a protease that is produced by synovial cells upon TNF-α stimulation, and its production is regulated by Ras (Abreu et al. [2009]). We analyzed MMP-3 levels and demonstrated that these affibody molecules also inhibited MMP-3 production (data not shown). These results indicate that affibody molecules binding to H-Ras or Raf-1 can inhibit inflammatory mediators or proteases, such as IL-6, PGE2, and MMP-3, which are crucial to RA pathogenesis.

Regarding the expression of affibody molecules in MH7A cells, we observed stronger expression of Z_ras122_ than other affibody proteins. In addition, Z_ras122_ protein levels increased, while Z_raf322_ and Z_ras521_ protein levels decreased between 24 and 48 h (Figure 1). These results might be related to the stability of the Z_ras122_ affibody protein afforded by the plasmid construct. Supporting this notion, we observed that Z_ras220_ and Z_ras521_ inhibited IL-6 production by 23.9% and 26.2% relative to the control vector treatment at 24 h, while these inhibitory effects were decreased to 17.8% and 10.1% at 48 h after TNF-α stimulation, respectively. In contrast, inhibition by Z_ras122_ was maintained up to 48 h after TNF-α stimulation (Figure 2A), suggesting increased stability of the affibody. With regard to PGE2 production, inhibition by all affibodies at 48 h increased compared with that at 24 h after TNF-α stimulation. Of interest, the timing of maximal inhibition was different for IL-6 and PGE2, but the underlying mechanism is still unknown. The difference could be attributed to the difference in the signal transduction cascade of these inflammatory molecules.

To determine whether affibody molecules inhibit signal transduction via ERK, we examined the phosphorylation of ERK1/2, a downstream molecule of Ras/Raf signaling. Z_ras220_ inhibited ERK1/2 phosphorylation in the early stage (16 h) after stimulation by TNF-α (Figure 3), although we did not observe distinct inhibition by other affibodies. This transient inhibition of ERK1/2 phosphorylation might be caused relatively by Z_ras220_ via interruption of the interaction between Raf-1 and MEK1/2 (Chen et al. [2001]). However, this inhibition on ERK1/2 phosphorylation could not be kept after 16-24 h, so that differences of phosphorylation among all affibody molecules here should be investigate more precisely in further study.

We utilized two different methods to deliver affibody molecules into synovial cells: 1) transfection of plasmids encoding the affibody molecules and 2) introduction of affibody proteins using a cell-penetrating peptide reagent. Protein-introduced affibody molecules exhibited stronger inhibition of IL-6 and PGE2 production than plasmid-expressed affibody molecules (Figures 2 and 4). Moreover, the inhibition resulting from the introduced proteins was less variable than that resulting from plasmid transfection. When cell proliferation was examined using plasmid-encoded affibody molecules, distinctive differences were not observed (Figure 5A). On the contrary, introduction of Z_raf322_ proteins partially inhibited cell proliferation (Figure 5B), suggesting that a plasmid-based expression system did not generate a sufficient concentration of affibody in MH7A cells to inhibit signal transduction. In the experiments using plasmids, cell proliferation was not observed at 24 (Figure 5A), while in the experiments using proteins, cell proliferation was observed at the same period (Figure 5B). This might be based on higher cytotoxicity of liposome compared with peptides used as a carrier (Suhorutsenko et al. [2011]). These results indicate that protein-introduced affibody molecules more effectively inhibited cytokine production and cell proliferation in synovial cells than plasmid-expressed affibody molecules. Although the effect on synovial cell proliferation was not strong, our results demonstrate that it is possible to inhibit synovial cell proliferation by selecting useful affibody clones and by improving transduction efficiency of affibody protein molecules.

Inhibition of IL-6 and PGE2 production caused by the protein-introduced affibodies varied. For example, at 48 h after TNF-α stimulation, inhibition of IL-6 production was greater than 40% for three affibodies (Z_ras122_, Z_ras220_, and Z_ras521_), whereas that of PGE2 was approximately 30% for two affibodies (Z_ras220_ and Z_raf322_). These results could be attributed to the fact that the PGE2 receptor, which couples with G proteins, is integrated into a positive feedback loop for PGE2 production (Nishimura et al. [2013]); thus, the effect of an affibody molecule on PGE2 production might be weaker than on IL-6. The differential effectiveness of each affibody molecule on synovial cell function might also be explained by differences in the amino acid sequences of the affibodies themselves. Each clone was selected using a phage display library of Z variants, and the amino acid sequences encoded by each clone are different.

In conclusion, we demonstrated that affibody molecules could inhibit cell proliferation and the production of inflammatory mediators by blocking the Ras/Raf signal transduction cascade. Although inhibition of cell function could be enhanced, and delivery methods should be examined further, we propose that these molecules are candidates to be investigated as a molecular-targeting therapy for RA. Studies are in progress to select useful affibody clones, to improve delivery methods of affibody protein molecules, and to verify the efficiency of affibodies in an RA model.

## Additional file

## Electronic supplementary material

Additional file 1: DNA sequence of affibody molecules in this study. (PDF 32 KB)

Below are the links to the authors’ original submitted files for images.

Authors’ original file for figure 1

Authors’ original file for figure 2

Authors’ original file for figure 3

Authors’ original file for figure 4

Authors’ original file for figure 5
