# Supplementary material for: Inhibitory effects of H-Ras/Raf-1-binding affibody molecules on synovial cell function
Source: AMB Express. 2014 Nov 11;4:82. doi: 10.1186/s13568-014-0082-3 (PMC4884024; doi:10.1186/s13568-014-0082-3)
Supplement: Supplementary file 1 — Additional file 1: DNA sequence of affibody molecules in this study. (PDF 32 KB) [file 13568_2014_82_MOESM1_ESM.pdf]

Additional file 1. DNA sequence of affibody molecules in this study.

Z_{ras122}

5′-GTAGATAACAAATTCAACAAAGAAACGGTGTGTGCGGTGGCGGAGATCGCGTGGTT
ACCTAACTTAAACAAGAGGCAAAAGAGGGCCTTCATCGAGAGTTTAGAGGATGACCCAA
GCCAAAGCGCTAACTTGCTAGCAGAAGCTAAAAAGCTAAATGATGCTCAGGCGCCGAAA
-3′

Z_{ras220}

5′-GTAGACAACAAATTCAACAAAGAAGCGTCGATTGCGAGTAAGGAGATCACGTATTT
ACCTAACTTAAACAGGCGGCAACGTGTGGCCTTCATCTGTAGTTTACTGGATGACCCAA
GCCAAAGCGCTAACTTGCTAGCAGAAGCTAAAAAGCTAAATGATGCTCAGGCGCCGAAA
-3′

Z_{ras521}

5′-GTAGATAACAAATTCAACAAAGAAAATAGGCCTGCGAGTATGGAGATCTTTTGTTT
ACCTAACTTAAACAATAAGCAAGTTCGTGCCTTCATCATGAGTTTACGTGATGACCCAA
GCCAAAGCGCTAACTTGCTAGCAGAAGCTAAAAAGCTAAATGATGCTCAGGCGCCGAAA
-3′

Z_{raf322}

5′-GTAGATAACAAATTCAACAAAGAAGTTAATCTTGCGGCGGATGAGATCTGGCTGTT
ACCTAACTTAAACAATCAGCAAGCGTGGGCCTTCATCACTAGTTTAAAGGATGACCCAA
GCCAAAGCGCTAACTTGCTAGCAGAAGCTAAAAAGCTAAATGATGCTCAGGCGCCGAAA
-3′

*Z_{ras122}, Z_{ras220} and Z_{ras521} were produced as dimmer using listed sequence, respectively.
