# Supplementary figures and images for: Inhibitory effects of H-Ras/Raf-1-binding affibody molecules on synovial cell function
Source: AMB Express. 2014 Nov 11;4:82. doi: 10.1186/s13568-014-0082-3 (PMC4884024; doi:10.1186/s13568-014-0082-3)

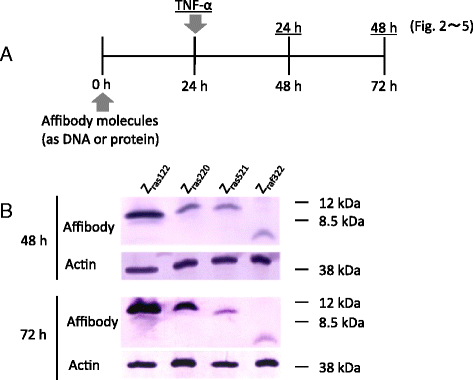

Supplement: Supplementary file 2 — Authors’ original file for figure 1 [file 13568_2014_82_MOESM2_ESM.gif]

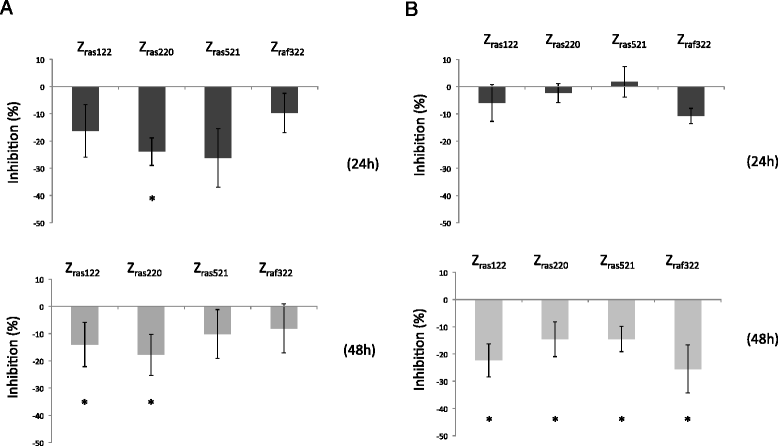

Supplement: Supplementary file 3 — Authors’ original file for figure 2 [file 13568_2014_82_MOESM3_ESM.gif]

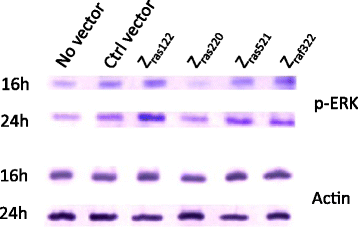

Supplement: Supplementary file 4 — Authors’ original file for figure 3 [file 13568_2014_82_MOESM4_ESM.gif]

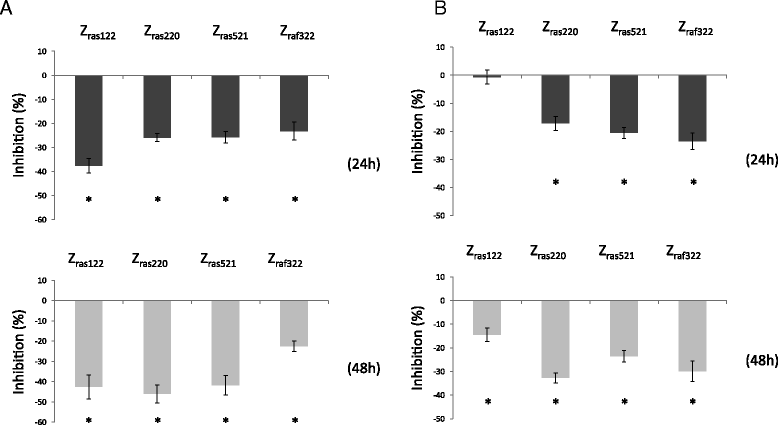

Supplement: Supplementary file 5 — Authors’ original file for figure 4 [file 13568_2014_82_MOESM5_ESM.gif]

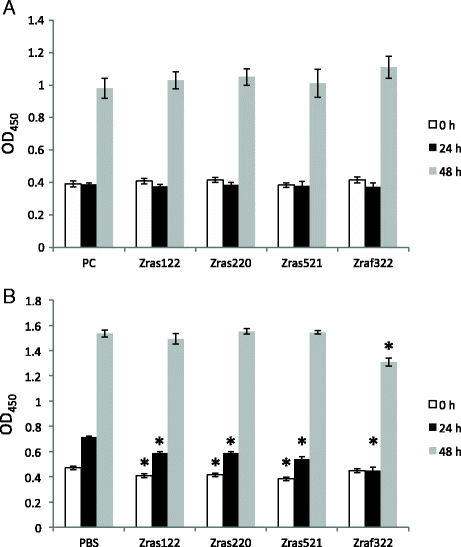

Supplement: Supplementary file 6 — Authors’ original file for figure 5 [file 13568_2014_82_MOESM6_ESM.gif]
